# Serotype-specific effectiveness against pneumococcal carriage and serotype replacement after ten-valent Pneumococcal Conjugate Vaccine (PCV10) introduction in Pakistan

**DOI:** 10.1371/journal.pone.0262466

**Published:** 2022-01-21

**Authors:** Muhammad Imran Nisar, Fyezah Jehan, Shahira Shahid, Sheraz Ahmed, Sadia Shakoor, Furqan Kabir, Aneeta Hotwani, Sahrish Muneer, Farah Khalid, Sajid Muhammad, Benjamin M. Althouse, Hao Hu, Cynthia G. Whitney, Asad Ali, Anita K. M. Zaidi, Saad B. Omer, Najeeha Iqbal

**Affiliations:** 1 Department of Pediatrics and Child Health, Aga Khan University, Karachi, Pakistan; 2 Department of Pathology and Laboratory Medicine, Aga Khan University, Karachi, Pakistan; 3 Bill & Melinda Gates Foundation, Seattle, WA, United States of America; 4 Emory University, Atlanta, GA, United States of America; 5 Yale Institute for Global Health, New Haven, CT, United States of America; Hamad Medical Corporation, QATAR

## Abstract

**Objective:**

Pakistan was one of the first South-Asian countries to introduce the ten-valent pneumococcal conjugate vaccine (PCV10) at the national level, using a 3+0 schedule without catchup, in 2013.

**Methods:**

From 2014–18, fifteen children <2 years old were recruited every week in Matiari, Sindh, and nasopharyngeal swabs were collected. The samples were cultured, and pneumococcus was further serotyped through multiplex PCR at the Aga Khan University Hospital as per the method described by the Centers for Disease Control and Prevention, USA.

**Results:**

Pneumococcus was detected in 2370/3140 (75%) children. Vaccine type (VT) and non-vaccine type (NVT) serotypes were carried by 379 and 1990 children. There was a significant decline in VT carriage (by 40.3%, p-value <0.001), whereas overall NVT carriage remained the same. The prevalence of VT serotypes 6B, 9V/9A, and 19F showed a significant decline by 58.8%, 79.3%, and 56%, respectively. The prevalence of NVT serotypes 19A, 21, and 10A increased by 70%, 33.3%, and 65.6%, respectively, whereas serotypes 13 and 9N/9L decreased by 53.4% and 51.8%, respectively. Serotype-specific vaccine effectiveness estimates that reached statistical significance were for 9V/9A (VE = 65.0, 95% CI 26.0–83.5%), 19F (VE = 55.3, 95% CI 15.5–76.4%) and for the vaccine related serotype 6A (VE = 28.4, 95% CI 0.9–48.2%).

**Conclusion:**

The emergence of NVT serotypes, primarily 19A replacing VT serotypes in this rural community, necessitates continuous monitoring of serotypes in the carriage and invasive disease to evaluate the utility of existing vaccine formulations.

## Introduction

*Streptococcus pneumoniae* (pneumococcus) is the leading cause of pneumonia, meningitis, and other serious infections, causing a high burden of morbidity and mortality in all ages worldwide [1]. The global estimate of deaths from pneumococcal infections was 318,000 in children <5 years in 2015, and 50% of these deaths occurred in developing countries. In Pakistan, the burden is specifically high, with an estimated 14,400 pneumococcal-related deaths in children less than five years of age in 2015, despite the introduction of vaccines [2].

Pneumococcus is usually spread by those who are colonized in their upper airway. Children are the main reservoirs and transmitters of pneumococcus. Colonization is mostly asymptomatic but may progress to severe disease in some cases [3]. There are more than 90 serotypes of pneumococcus known so far, but only a few are responsible for causing most invasive pneumococcal disease (IPD) [1, 4, 5]. More than half of the IPD cases worldwide are caused by seven serotypes (1, 5, 6A, 6B, 14, 19F, and 23F) [6]. Carriage and transmission of these serotypes can be reduced by vaccination with the existing conjugate pneumococcal vaccines (PCVs). The 7-valent vaccine (PCV7, Prevnar, Pfizer) was the first available conjugate vaccine and was introduced in the U.S. and a limited number of other countries beginning in the year 2000; PCV7 contained serotypes 4, 6B, 9V, 14, 18C, 19F, and 23F. Serotypes covered by the PCV7 formulation were responsible for 80% of IPD occurring in children less than 5 years in North America and at least 50% of IPD in other regions, but PCV7 covered only 30% of IPD in Asia, where serotypes 1 and 5 caused significant disease burden [6, 7]. PCV7 was then replaced by higher valent vaccines, PCV10 and PCV13, by the end of the decade. PCV10 (Synflorix, GlaxoSmithKline) covered three additional serotypes (1, 5, and 7F) and PCV13 (Prevnar13, Pfizer) added three serotypes (3, 6A, and 19A) to the PCV10 serotypes. Of these types, 6A and 19A are considered potentially cross-reactive to serotypes 6B, 6C and 19F and are responsible for 8%–15% of the IPD burden [6]. Both PCV10 and PCV13 have a greater serotype coverage. With assistance from Gavi, the Vaccine Alliance, Pakistan introduced PCV10 in its Expanded Program on Immunization (EPI) in early 2013. It was the first country in South Asia to do so, using a schedule of three doses given at 6, 10 and 14 weeks of life and no catchup immunization (3+0 schedule) [8].

PCVs reduce pneumococcal transmission in the community by reducing the vaccine-type serotypes in the nasopharynx. However, non-vaccine-type pneumococci rapidly colonize this vacated ecological niche, a process known as serotype replacement. Although the serotypes most frequently carried in the nasopharynx are not always the ones responsible for invasive disease. Monitoring trends in changing epidemiology of serotypes is still pivotal for assessing the benefit of PCVs. In addition, because pneumococcal transmission within a population is mainly from colonized persons, measurement of prevalent serotypes in the nasopharynx can be used to evaluate vaccine effectiveness for reducing circulation of vaccine-type serotypes. We have previously reported a high burden of pneumococcal carriage (80% in children aged 3–11 months) before the introduction of the vaccine, with a carriage rate of 26.7% for PCV10 serotypes [9]. Here we present serotype distribution of various VT and NVT serotypes in the nasopharynx of children <2 years after PCV10 introduction, vaccine effectiveness against individual serotypes, and change in serotype distribution.

## Methods

This study was carried out in Khyber and Shah Alam Shah Jee Wasi, two rural union councils of Matiari, Sindh, between October 2014 and September 2018. These union councils have a total registered population of around 88,739. The study site was selected because of a pre-PCV10 introduction carriage survey done in the same population in 2012–13. In that survey, 225 children aged from 3–12 months were enrolled between Feb-March 2013. All the specimen collection and laboratory methods were similar to the current study. During 2014–2018, children were identified through random selection from a continuously updated line listing of all children <2 years of age. Approximately 15 children were enrolled every week from the line-listing after written informed consent was obtained from their guardians. Each child was enrolled only once in the study. All children under the age of 2 years residing in the area for at least six months with no nose and throat abnormalities or severe illness requiring hospitalization were eligible to be enrolled in the study.

Demographic and clinical information, including past hospitalization and outpatient visits, data on vaccination, and potential risk factors for the pneumococcal carriage, was collected by study staff. Data on vaccination was recorded based on verbal recall or vaccination cards where available. If the vaccination card was unavailable, the mothers were probed for PCV10 vaccination by confirming the age at which their child got vaccinated and the site where it was administered. As per local practice by vaccinators, PCV10 is injected in the left thigh and the pentavalent vaccine in the right thigh. Data were collected by trained study personnel on a custom-built data capturing application for android phones. The questionnaires were designed in.xml format using Open Data Kit (ODK) software. The data was transferred at the end of the day to a server located at the Aga Khan University’s Data Management Unit in Karachi through the internet. A brief clinical exam, including temperature measurement, respiratory rate, and observation for chest wall indrawing, was also performed.

### Laboratory procedures

Nasopharyngeal specimens were collected and transported at 2–8˚C from the field site to the infectious diseases research laboratory (IDRL) in Karachi within 8 hours using established World Health Organization’s (WHO) consensus methods [10].

Upon arrival in IDRL, samples were immediately stored upright at -80°C until further processing. Batches of 20–40 samples were thawed, cultured, and sub-cultured, as mentioned in the CDC protocol [11]. Briefly, an aliquot of the thawed STGG mixture was enriched in Todd Hewitt broth supplemented with rabbit serum (20%) and yeast extract (0.5%) and subcultured onto TSA medium with 5% sheep blood. After 18–24 hours of incubation, plates were examined for the presence of alpha-hemolytic colonies. Identification was confirmed by susceptibility to optochin and bile solubility testing. Isolates were then tested using conventional multiplex PCR to identify different pneumococcal serotypes [12]. DNA extraction was performed by boiling the bacterial culture for 10 minutes and centrifugation for another 10 minutes; the resulting supernatant was collected in a sterile microcentrifuge tube. The remaining DNA extract was used for PCR techniques. The *cpsA* gene served as an internal positive control in all multiplex reactions. DNA of 2μl was added to the PCR master mix containing nuclease-free water, 2X Qiagen multiplex PCR buffer, Qiagen Q solution, and 25 μM working stock of primers. Amplification was carried out in an Eppendorf Master Cycler Gradient with the specific temperature profile, and the amplified PCR products were stained and read under BioRad Gel Doc imager. Serotypes were detected through sequential multiplex conventional PCR, which was further confirmed by monoplex PCR, using the same conditions, to avoid misidentification due to non-specific bands. Serogroup 6 was additionally differentiated into serotypes 6A, 6B, 6C, and 6D by the same method used by Jin et al. with some modifications as mentioned earlier [13]. Only one serotype per specimen was identified. Pneumococcal serotype controls added in each reaction were obtained from CDC streptococcal lab. The non-typeable products were confirmed as pneumococci using *lytA* real-time PCR as described by Carvalho et al. [12].

*Streptococcus pneumoniae* ATCC 49619 and *Enterococcus faecalis* ATCC 29212 strains were used for quality control in the optochin susceptibility and bile solubility reactions. Further details are provided in separate papers [14, 15].

### Statistical analyses

Vaccine Type (VT) carriage was defined as presence of any of the 10 serotypes included in PCV10 (i.e. serotypes 1, 4, 5, 6B, 7F, 9V, 14, 18C, 19F, and 23F). Only one serotype was identified per sample. VT carriage was calculated as the number of samples positive for VT serotypes as a proportion of all study participants. Cross-reactive serotypes like 6A and 19A and all other serotypes besides the above ten were classified as non-vaccine type (NVT). PCV13 serotypes carriage was defined as the presence of any of the serotypes included in the 13-valent PCV (i.e., serotypes 1, 4, 5, 6B, 7F, 9V, 14, 18C, 19F, 23F, 3, 6A, and 19A). A study year ran from October of one year through September of next year. We describe the carriage rate by the number of vaccine doses and study year. We describe serotype distribution by year of the study, looking at trends for various VT and NVT serotypes individually and grouped.

For the period 2014–2018, we calculated vaccine effectiveness (VE) for specific serotypes. We separately calculated VE for 3, 2, and 1 dose of PCV10 using the following formulae:

$$1-\left(\frac{prevalence\;of\;a\;serotype\;in\;those\;who\;received\;3\;doses\;during\;2014-2018\;period\;}{prevalence\;of\;a\;serotype\;in\;those\;who\;received\;0\;dose\;during\;2014-2018\;period}\right)$$

Statistical significance of VE was established if the lower limit of the 95% CI around VE was above 0 (zero); alpha was set at 0.05. All analyses were done using Stata version 15.0 and Microsoft Excel 2017.

### Ethical approval

Ethical approval was obtained from Aga Khan University’s Ethical Review Committee. Written informed consent was obtained from all caretakers before commencing enrollment in all surveys.

## Results

### Characteristics of study participants

Detailed demographic and clinical characteristics have been described previously [15]. A total of 3,140 children under two years were enrolled between October 2014 and September 2018. The mean age was 10.5 months, half of the enrolled children were male, and most primary caretakers and half of the primary wage earners had no education. The median household size was 8; one-third of children were exposed to environmental tobacco smoke. A history of cough, runny nose, and fever in the past two weeks was common. Six percent had fever at enrollment, 7% had tachypnea, and 1.5% had chest wall indrawing. According to verbal reports or card verification, the proportion of fully vaccinated children (who received all three PCV10 doses) increased from 41.0% to 68.4% over the study period. Vaccination status was documented for 2091 (66.6%) of children.

### Carriage prevalence

**Fig 1:**
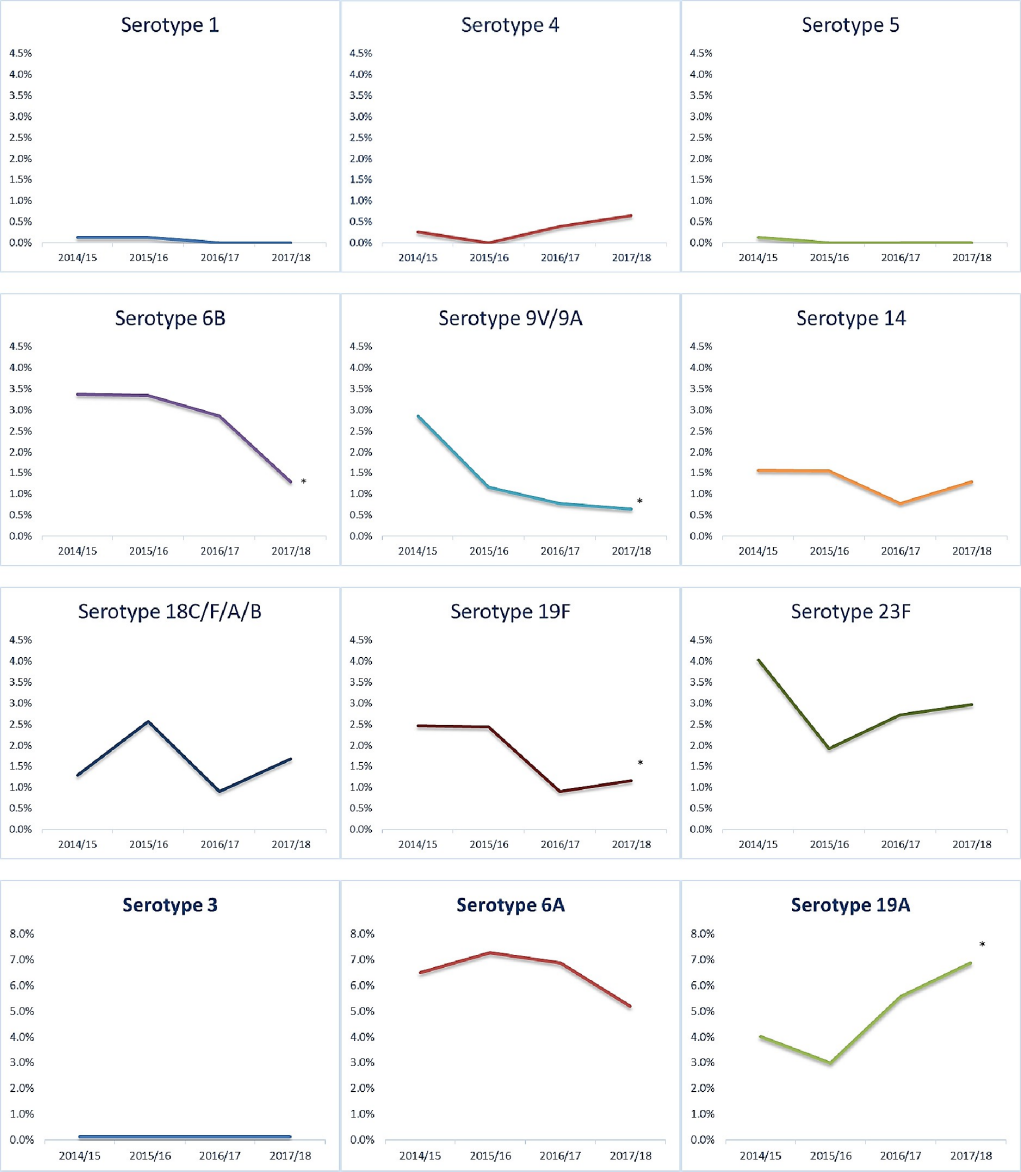
Individual serotype carriage rates over the study period.

We detected pneumococcus in the nasopharyngeal samples of 2,369 children; serotypes were determined for 2154 isolates. Serotypes of 215 (9%) isolates were not identified by the methods used and were classified as non-typeable. Nine VT serotypes were detected; 7F was not be identified (Table 1 and Fig 1). Pneumococcal colonization decreased from 80.8% in the first year of the study to 72.8% in the final year (p-value for trend = 0.001). The prevalence of VT serotypes significantly decreased by 71.3%, from 16.1% in the first year to 9.6% in the fourth year (p-value for trend <0.001). NVT carriage remained unchanged. VT carriage was lower among children who received three PCV10 doses compared to unvaccinated children (those who received 0 dose) (9.9% and 14.8%, p = 0.001) [15].

**Table 1:** Serotype specific carriage rate from 2014–18 in Matiari, Pakistan.

Serotype	2013 (Baseline)^α^	2014–15	2015–16	2016–17	2017–18	p-value
	N = 225	N = 771	N = 780	N = 779	N = 810	
PCV10-types	n (%)	n (%)	n (%)	n (%)	n (%)	
**1**	-	1 (0.1)	1 (0.1)	0 (0)	0 (0)	0.562
**4**	-	2 (0.3)	0 (0)	3 (0.4)	5 (0.6)	0.174
**5**	-	1 (0.1)	0 (0)	0 (0)	0 (0)	0.380
**6B** *	7 (3.1)	26 (3.4)	26 (3.3)	23 (3.0)	11 (1.4)	0.043
**9V/9A** *	9 (4.0)	22 (2.9)	9 (1.2)	6 (0.8)	5 (0.6)	<0.001
**14**	8 (3.6)	12 (1.6)	12 (1.5)	7 (0.9)	11 (1.4)	0.646
**18C/18F/18B/18A**	5 (2.2)	10 (1.3)	20 (2.6)	7 (0.9)	13 (1.6)	0.057
**19F** *	8 (3.6)	19 (2.5)	19 (2.4)	8 (1.0)	9 (1.1)	0.033
**23F**	23 (10.2)	31 (4.0)	15 (1.9)	21 (2.7)	24 (3.0)	0.102
**PCV-13 specific types**						
**3**	-	1 (0.1)	1 (0.1)	1 (0.1)	1 (0.1)	1.0
**6A**	19 (8.4)	50 (6.5)	56 (7.2)	53 (6.8)	42 (5.2)	0.391
**19A** *	16 (7.1)	31 (4.0)	23 (2.9)	43 (5.5)	55 (6.8)	0.002
**NVT serotypes**						
**13** *	1 (0.4)	33 (4.3)	20 (2.6)	18 (2.3)	16 (2.0)	0.027
**20**	-	0 (0)	1 (0.1)	4 (0.5)	3 (0.4)	0.176
**21** *	3 (1.3)	9 (1.2)	9 (1.2)	9 (1.2)	23 (2.8)	0.013
**31**	-	1 (0.1)	2 (0.3)	2 (0.3)	1 (0.1)	0.874
**34**	2 (0.9)	19 (2.5)	27 (3.5)	16 (2.1)	15 (1.9)	0.167
**10A** *	7 (3.1)	25 (3.2)	44 (5.6)	55 (7.1)	43 (5.3)	0.010
**10F/10C/33C**	1 (0.4)	15 (1.9)	11 (1.4)	8 (1.0)	8 (1.0)	0.319
**11A/11D**	2 (0.9)	30 (3.9)	30 (3.8)	32 (4.1)	39 (4.8)	0.752
**12F/12A/44/46**	1 (0.4)	6 (0.8)	9 (1.2)	3 (0.4)	2 (0.3)	0.099
**15B/15C**	6 (2.7)	43 (5.6)	35 (4.5)	55 (7.1)	36 (4.4)	0.072
**15F/15A** *	4 (1.8)	12 (1.6)	3 (0.4)	15 (1.9)	8 (0.1)	0.031
**16F**	1 (0.4)	21 (2.7)	17 (2.2)	22 (2.8)	13 (1.6)	0.345
**17F**	6 (2.7)	15 (1.9)	11 (1.4)	9 (1.2)	17 (2.1)	0.414
**22F/22A**	1 (0.4)	7 (0.9)	3 (0.4)	7 (0.9)	2 (0.2)	0.199
**23A**	1 (0.4)	7 (0.9)	6 (0.8)	9 (1.2)	11 (1.4)	0.668
**23B**	5 (2.2)	20 (2.6)	21 (2.7)	9 (1.2)	18 (2.2)	0.141
**24F/24A/24B**	6 (2.7)	5 (0.6)	6 (0.8)	3 (0.4)	10 (1.2)	0.264
**33F/33A/37**	-	6 (0.8)	6 (0.8)	10 (1.3)	8 (1.0)	0.697
**35A/35C/42**	-	4 (0.5)	10 (1.3)	8 (1.0)	9 (1.1)	0.464
**35B**	5 (2.2)	28 (3.6)	20 (2.6)	28 (3.6)	24 (3.0)	0.567
**35F/47F**	1 (0.4)	5 (0.6)	0 (0.0)	2 (0.3)	1 (0.1)	0.064
**38/25F/25A**	-	5 (0.6)	2 (0.3)	5 (0.6)	7 (0.9)	0.471
**6C**	2 (0.9)	11 (1.4)	3 (0.4)	5 (0.6)	11 (1.4)	0.082
**6D**	8 (3.6)	10 (1.3)	11 (1.4)	7 (0.9)	9 (1.1)	0.799
**7C/7B/40** *	5 (2.2)	10 (1.3)	25 (3.2)	7 (0.9)	10 (1.2)	0.001
**9N/9L** *	6 (2.7)	21 (2.7)	11 (1.4)	4 (0.5)	4 (0.5)	<0.001
**2**	-	1 (0.1)	0 (0.0)	0 (0.0)	0 (0.0)	0.380
**8**	-	3 (0.4)	1 (0.1)	1 (0.1)	1 (0.1)	0.552
**All PCV10-types** * **(VT)**	60 (26.7)	124 (16.1)	102 (13.1)	75 (9.6)	78 (9.6)	<0.001
**Non-PCV10-types(NVT)**	165 (73.3)	499 (64.7)	473 (60.6)	508 (65.2)	510 (63.0)	0.927
**Non-PCV13-types** *	130 (57.7)	417 (54.0)	415 (53.2)	389 (49.9)	393 (48.5)	<0.001
**Non-typeables**	8 (3.6)	45 (5.8)	49 (6.3)	58 (7.4)	63 (7.8)	0.083

*p-value for trend <0.05

^α^ Pre-vaccine introduction data (2013) is not included in the trend analysis.

Vaccine Type (VT) carriage includes any of the 10 serotypes in PCV10 (serotypes 1, 4, 5, 6B, 7F, 9V, 14, 18C, 19F, and 23F). Nonvaccine type (NVT) carriage was defined as the presence of all other serotypes besides PCV10 specific serotypes, including vaccine-related and non-typeable strains. PCV13 serotypes include serotypes 1, 4, 5, 6B, 7F, 9V, 14, 18C, 19F, 23F, 3, 6A and 19A. Serotypes isolated only at baseline and not isolated between 2014 and 2018 are omitted from the table.

### Serotype-specific changes

Fig 1 shows the change in carriage rate per year of individual VT serotypes and the three additional serotypes specific to PCV13 (3, 6A and 19A). The most prevalent serotypes reported were: 6A (n = 201), 19A (n = 152), 15B/15C (n = 169), 10A (n = 167), 11A/11D (n = 131) and 35B (n = 100) (Table 1). As seen from the panel (Fig 1), a statistically significant decline was observed in VT serotypes, 6B (from 3.4 to 1.4%, p value for trend = 0.043), 9V/9A (from 2.9 to 0.6%, p value for trend<0.001), and 19F (from 2.4 to 1.1%, p value for trend = 0.033). On the other hand, there was a significant increase in the carriage rate of NVT serotypes 19A, from 4.0 to 6.8%, (p-value for trend = 0.002). Among other ten most common NVT serotypes, a significant increase in carriage was seen for serotype 21 from 1.2% to 1.6% (p-value for trend = 0.013), 10A from 3.2% to 5.3% (p-value for trend = 0.010; Table 1) whereas serotypes 13 decreased from 4.3% to 2.0% (p-value for trend = 0.027), and 9N/9L from 2.7% to 1.3% (p-value for trend = <0.001).

The carriage rates of VT serotypes 4, 9V/9A, and 19F declined significantly in children who received all three doses of PCV10. Other VT serotypes also showed a decreasing trend, although the changes were not significant. Among the NVT serotypes, carriage of 19A (5.5 vs 3.4% p-value 0.040) and 11A/11D (4.6 vs 2.0%, p-value 0.005) was significantly higher in children vaccinated with 3 doses than among non-vaccinated children, whereas the converse was true for 6A (6.0 vs 8.3%, p-value 0.044; Table 2).

**Table 2:** Serotype-specific carriage rate by vaccination status.

Serotype	Vaccinated (3 doses)	Unvaccinated (0 dose)	p-value
	N = 1810	N = 588	
**PCV10-types**	n (%)	n (%)	
**1**	1 (0.1)	1 (0.2)	0.402
4*	3 (0.2)	4 (0.7)	0.045
**6B**	37 (2.0)	20 (3.4)	0.061
**9V/9A** *	14 (0.8)	13 (2.2)	0.004
**14**	27 (1.5)	4 (0.9)	0.130
**18C/18F/18B/18A**	25 (1.4)	9 (1.5)	0.790
**19F** *	22 (1.2)	16 (2.7)	0.011
**23F**	51 (2.8)	20 (3.4)	0.468
**Additional PCV-13 specific types**			
**3**	2 (0.1)	1 (0.2)	0.723
**6A** *	108 (6.0)	49 (8.3)	0.044
**19A** *	100 (5.5)	20 (3.4)	0.040
**NVT serotypes**			
**13**	44 (2.4)	17 (2.9)	0.538
**20**	5 (0.3)	0 (0)	0.202
**21**	30 (1.7)	9 (1.5)	0.833
**31**	3 (0.2)	0 (0)	0.323
**34**	48 (2.7)	14 (2.4)	0.719
**10A**	106 (5.9)	23 (4.0)	0.069
**10F/10C/33C**	19 (1.1)	9 (1.5)	0.346
**11A/11D** *	84 (4.6)	12 (2.0)	0.005
**12F/12A/44/46**	12 (0.7)	5 (0.9)	0.638
**15B/15C**	110 (6.1)	27 (4.6)	0.178
**15F/15A**	24 (1.3)	8 (1.4)	0.949
**16F**	43 (2.4)	13 (2.2)	0.818
**17F**	33 (1.8)	6 (1.0)	0.181
**22F/22A**	11 (0.6)	4 (0.7)	0.846
**23A**	22 (1.2)	6 (1.0)	0.702
**23B**	37 (2.4)	16 (2.7)	0.332
**24F/24A/24B**	16 (0.9)	4 (0.7)	0.637
**33F/33A/37**	19 (1.1)	6 (1.0)	0.952
**35A/35C/42**	19 (1.1)	5 (0.9)	0.673
**35B**	57 (3.2)	13 (2.2)	0.240
**35F/47F**	4 (0.2)	1 (0.2)	0.814
**38/25F/25A**	13 (0.7)	1 (0.2)	0.130
**6C**	21(1.2)	6 (1.0)	0.780
**6D**	18 (1.0)	10 (1.7)	0.166
**7C/7B/40**	29 (1.6)	11 (1.9)	0.659
**9N/9L** *	9 (0.5)	23 (3.9)	<0.001
**2**	1 (0.1)	0 (0)	0.569
**8**	3 (0.2)	1 (0.5)	0.146
**All PCV10-types (VT)** *	180 (9.9)	87 (14.8)	0.001
Non-PCV10-types (NVT)*	1194 (66.0)	351 (59.7)	0.006
**Non-PCV13-types**	984 (54.3)	281 (47.7)	0.450

*p-value less than 0.05.

### Vaccine effectiveness

Table 3 shows the carriage rate of VT serotypes in children who received 0, 1, 2, or 3 doses of the vaccine. In children receiving 3 doses of PCV10, VE against all VT serotypes was 32.8% (95% CI 14.7–47.0); it was 11.8% (95% CI -21.5–36.1) for doses and -17.5% (95% CI-58.5–12.9) for one dose.

**Table 3:** The carriage rate of VT serotypes by number of doses of vaccine.

	Number of doses			
	0 dose	1 dose	2 doses	3 doses
	N = 588	N = 351	N = 391	N = 1810
**VT detected (%)**	87 (14.8)	61 (17.4)	51 (13.0)	180 (9.9)
**VT not detected (%)**	501 (85.2)	290 (82.6)	340 (87.0)	1630 (90.1)

**Fig 2:**
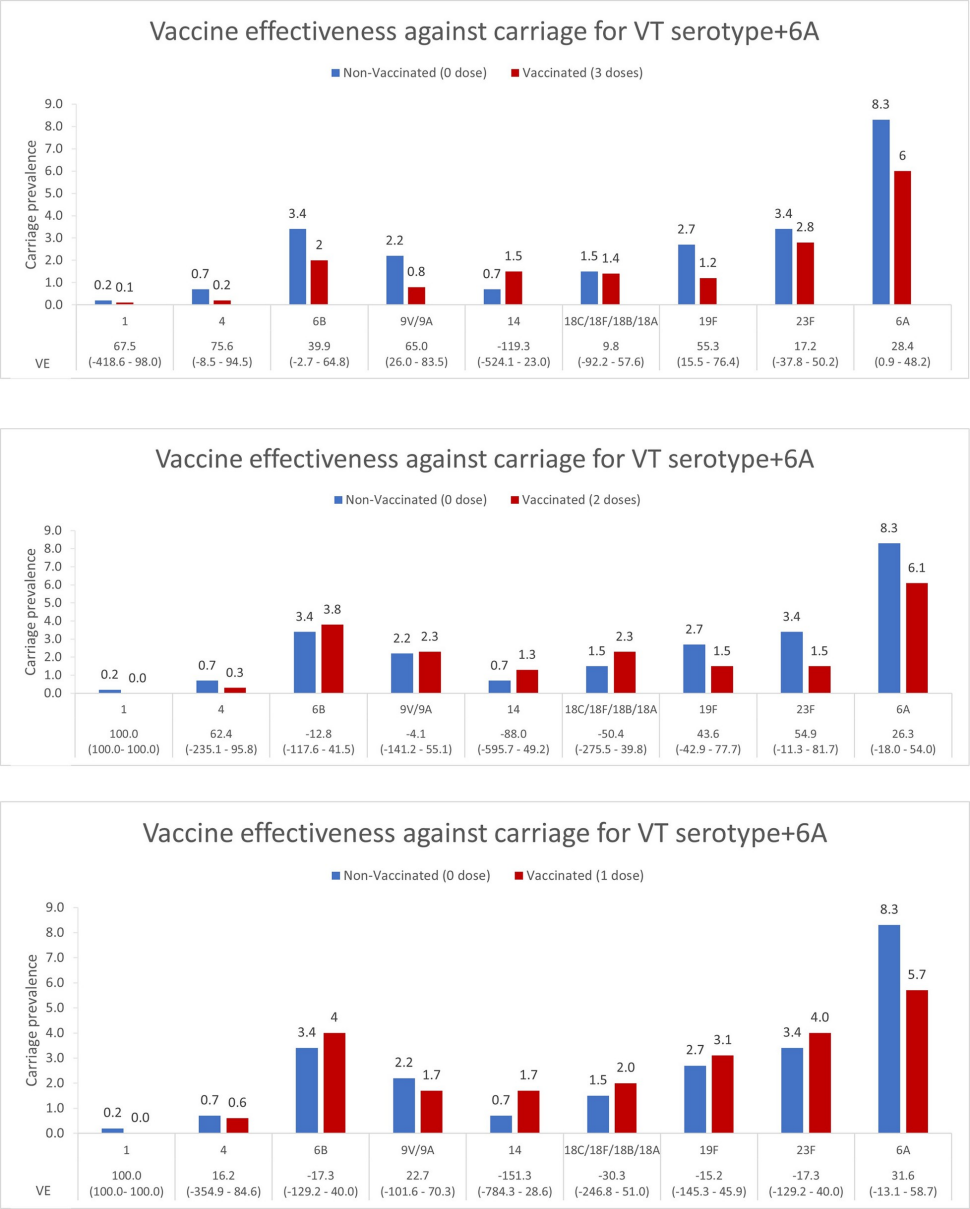
Vaccine effectiveness against specific serotypes, (a) 0 dose vs 3 doses, (b) 0 dose vs 2 doses and (c) 0 dose vs 1 dose.

For 3 doses versus 0 dose-comparison, VE estimates for serotypes 9V/9A (VE = 65.0, 95% CI 26.0–83.5%), 19F (VE = 55.3, 95% CI 15.5–76.4%) and 6A (VE = 28.4, 95% CI 0.9–48.2%) reached statistical significance (Fig 2A). VE estimates for none of the serotypes reached statistical significance when comparison was made for one or two PCV doses against no dose (Fig 2B and 2C).

## Discussion

To the best of our knowledge, this is the first study evaluating VE of PCV10 against individual serotypes and change in serotype distribution in the backdrop of increased vaccine coverage from 2014 to 2018. Overall, VE was 32.8% with three doses of PCV10. This is most likely an underestimate as unvaccinated children also benefitted from the indirect effect of the vaccine. We could not directly compare the ‘true’ unvaccinated population from the pre-introduction survey as the age groups were different [9]. Kokko et al. report global estimates of VE against pneumococcal carriage of all vaccine serotypes together to be between 44–65% [17]. Since we were not powered to detect VE for individual serotypes, our analysis for that was exploratory. VE estimates were statistically significant for only serotypes 9V/9A, 19F, and 6A when comparison was made between children who received three doses versus those who received zero dose.

A complete regimen of three doses conferred greater protection than either one or two doses. This is consistent with the literature. A cross-sectional survey in Brazil showed a VE of 36% (95% CI 4–57) for two doses as compared to 44% (95% CI 14–64) for three doses [18]. A systematic review and meta-analysis by Nicholls et al. of 16 trials of PCVs among 14,776 children found no evidence of protection against VT colonization from a single dose, but a two dose and three dose regimen showed a VE of 19% and 33%, respectively [19]. However, none of these studies had the statistical power for VE against individual serotypes.

In the fully vaccinated group, i.e., those who received all three doses of PCV10, carriage of VT serotypes 4, 9V/9A, and 19F declined significantly. There was a non-statistically significant increase in the carriage of serotype 14 and a non-statistically significant decrease in the carriage of other VT serotypes. A significant reduction was seen for serotype 6A and a significant increase for serotype 19A in the fully vaccinated group (3 doses). Although the increase in serotype 19A carriage suggests a potential for replacement disease, pre- and post-PCV studies of the IPD from our region do not show 19A as a leading cause [20]. Other studies have shown mixed results regarding the PCV13 specific serotypes. A study in Mozambique showed the colonization prevalence for the three PCV13-specific serotypes (3, 6A, 19A) increased by 40% from 12.4% to 20.7% three years after introducing PCV10 [21]. In contrast, Brazil, which introduced a PCV10 3 + 1 dose schedule with a catch-up for children under two years of age, showed no increase in carriage of the additional three serotypes in PCV13 [22]. A clinical trial from Finland also showed a reduction of serotype 19A carriage only in children aged 18–22 months who had received a 3+1 schedule [23]. However, another study in Israel noted reductions in both serotypes 6A and 19A post-introduction of PCV10 [24]. This could be due to cross-reactivity that has been reported between serotype 6B and serotype 6A in various studies [25, 26].

Although overall nasopharyngeal carriage declined throughout our study, VT carriage showed a significant reduction from the pre-vaccine era, and carriage of NVT serotypes increased, particularly 19A, 21,10A, and 7C/7B/40. Other studies from various parts of the world, including Mozambique, Kenya, Israel, and Brazil, have also shown a significant increase in NVT carriage rate after introducing PCV10 [21, 22, 24, 27]. However, in Fiji and South Africa, a decline was observed for NVT carriage [28, 29].

Our biggest strength was prospectively collected data on a large sample in a community setting over four years. We followed WHO and CDC standard protocols for the processing of samples. Our study was done in a rural part of Pakistan, yet it is still generalizable to large parts of south-Asia. Our investigation has some limitations, however. For example, we did not look for carriage of multiple serotypes or measure carriage density. Statistical power was limited in our estimates of vaccine effectiveness, as there were fewer vaccinated children. Our study does not address how doses were spaced within the 3+0 schedule. There could be potential bias due to unmeasured confounders, temporal factors, and misclassification due to verbal recall of vaccination status. Our data was restricted to the children less than two years of age group, precluding conclusions of the effect of vaccination in other age groups.

We could not directly compare with the pre-introduction baseline carriage survey as the age groups were different. However, a significant decline was seen for VT serotypes from 26.7% in 2013 to 9.6% in 2018 [9].

## Conclusion

We conclude that in the post PCV10 era from 2014 to 2018, the overall NVT carriage remained constant. However, a significant rise was seen in the carriage rate of serotypes 19A, 21, and 10A. VT carriage declined during the same period. Continual follow-up is required to track changes in serotype distribution in both carriage and disease to guide future vaccine formulations.
